# Molecular identification of *Actinomadura madurae* isolated from a patient originally from Algeria; observations from a case report

**DOI:** 10.1186/s12879-020-05552-z

**Published:** 2020-11-11

**Authors:** Arezki Izri, Mohanad Aljundi, Typhaine Billard-Pomares, Youssouf Fofana, Anthony Marteau, Theo Ghelfenstein Ferreira, Sophie Brun, Frederic Caux, Mohammad Akhoundi

**Affiliations:** 1 Parasitology-Mycology Department, Avicenne Hospital, AP-HP, Sorbonne Paris Nord University, Bobigny, France; 2 Dermatology Department, Avicenne Hospital, AP-HP, Sorbonne Paris Nord University, Bobigny, France; 3 Bacteriology Department, Avicenne Hospital, AP-HP, Sorbonne Paris Nord University, Bobigny, France

**Keywords:** Madura foot, Actinomycetoma, White grains, Molecular identification, Imaging; case report

## Abstract

**Background:**

Mycetoma is a chronic granulomatous subcutaneous infection caused by anaerobic pseudofilamentous bacteria or fungi. It is commonly prevalent in tropical and subtropical countries. Men are more susceptible to the disease due to greater participation in agricultural works. Mycetoma commonly involves lower extremities, wherein untreated cases lead to aggressive therapeutic choices, such as amputation of the affected body organs and consequently lifelong disability.

**Case presentation:**

In this report, we present the rare case of a 58-year-old man, originally from Algeria with a left foot chronic tumefaction of 5 years. In the initial clinical examination, mycetoma was diagnosed based on tumefaction and the presence of multiple sinuses with the emission of white grains. The latter was observed via direct examination. The histopathological analysis demonstrated an actinomycetoma caused by bacteria, as the etiological agent. Imaging showed a bone involvement with osteolysis at the levels of 2nd to 4th metatarsal diaphysis. The mycological and bacterial cultures were both negative. For an accurate diagnosis, the obtained grains were subjected to molecular analysis, targeting the 16S-rDNA gene. Molecular identification yielded *Actinomadura madurae* as the causal agent, and 800/160 mg of trimethoprim/sulfamethoxazole was prescribed twice a day for 1 year, as a treatment.

**Conclusion:**

Considering low information about this disease, especially in non-endemic areas, it is of high importance to enhance the knowledge and awareness of clinicians and healthcare providers, in particular in the countries with immigration issues.

## Background

Mycetoma is a chronic subcutaneous tissue infection caused by anaerobic pseudofilamentous bacteria (actinomycetoma) or fungi (eumycetoma). It is commonly observed in tropical and subtropical regions in a zone, known as the “Mycetoma belt” with dry and arid climates. India, Sudan, Somalia, Senegal, Yemen, Mexico, Venezuela, Colombia, and Argentina bear the most cases of disease burden [1]. The cases outside this zone are uncommon and usually imported by immigrants. Despite multiple cases reported worldwide, the incidence and prevalence of the disease remain underestimated [2]. Mycetoma is more prevalent in people between 20 and 40 years old, with a low socioeconomic level, and is more common in men rather than women (3:1) [3]. Mycetoma occurs mostly in the lower extremities of the body, particularly in the feet but other parts of the body can also be involved [4]. Although mycetoma is mostly a painless infection leading to delayed medical consultation, it can be painful in case of secondary bacterial infection [5, 6]. It is usually characterized by single or multi-fistulized pseudotumor and the emission of grains. The grains can vary in size, color, and consistency, depending on the etiological species [3].

Considering almost similar clinical manifestations appearing in actinomycetoma and eumycetoma and the differences in their treatments, accurate identification of causal microorganisms is crucial [7]. Furthermore, due to significant clinical presentations and complexity in therapeutic implications, the disease diagnosis in the early stages is of high importance. Delay in diagnosis may lead to the aggressive therapeutic selections, like amputation of affected body members, and consequently lifelong disability [8]. The diagnosis is mainly based on clinical characteristics and microbiological identification of the causative agent [3].

## Case presentation

A 58-year-old man with a chronic tumefaction of the left foot was referred due to suspicion of mycetoma. The patient was a restaurant-worker, originally from Algeria who was inhabited in France since 2001, with regular travel to his homeland but not to the known classical endemic areas. According to the patient, he was living in a rural region in Kabylie (north-east of Algeria) and suffered from left foot tumefaction for 5 years. He was initially hospitalized in 2017, in a health center in Paris. The initial medical check-up was performed including imaging, microbiological and histopathological examinations. The clinical diagnosis was made by a classical triad of tumefaction (32 cm perimeter of left foot comparing to 26 cm for right foot), presence of multiple sinuses, and emission of white grains. *Staphylococcus aureus,* and an anaerobic Gram-positive bacillus-like bacterium similar to *Actinomyces* sp., were identified, using classical microbiological culture with no species-specific identification. Significant infiltration of periosseous soft tissues and an osteolytic aspect of 2nd and 4th metatarsal with multiple geodes were observed by radiology and MRI. Echography did not reveal superficial thrombophlebitis in association with this tumefaction. Therapy was initialized, using amoxicillin (6 g/day for 4 months) as the first-line therapeutic choice during initial hospitalization, in 2017. Due to no favorable evolution, his treatment was left incomplete.

**Fig. 1:**
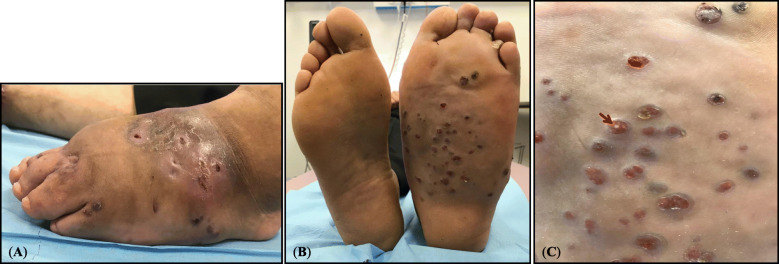
**a** Presence of mycetoma grains and sinuses on the top side of the left foot; **b** Left foot mycetoma presenting grains and sinuses comparing to healthy right foot; **c** Close vision of the grains and sinuses (red arrow) on the left foot

**Fig. 2:**
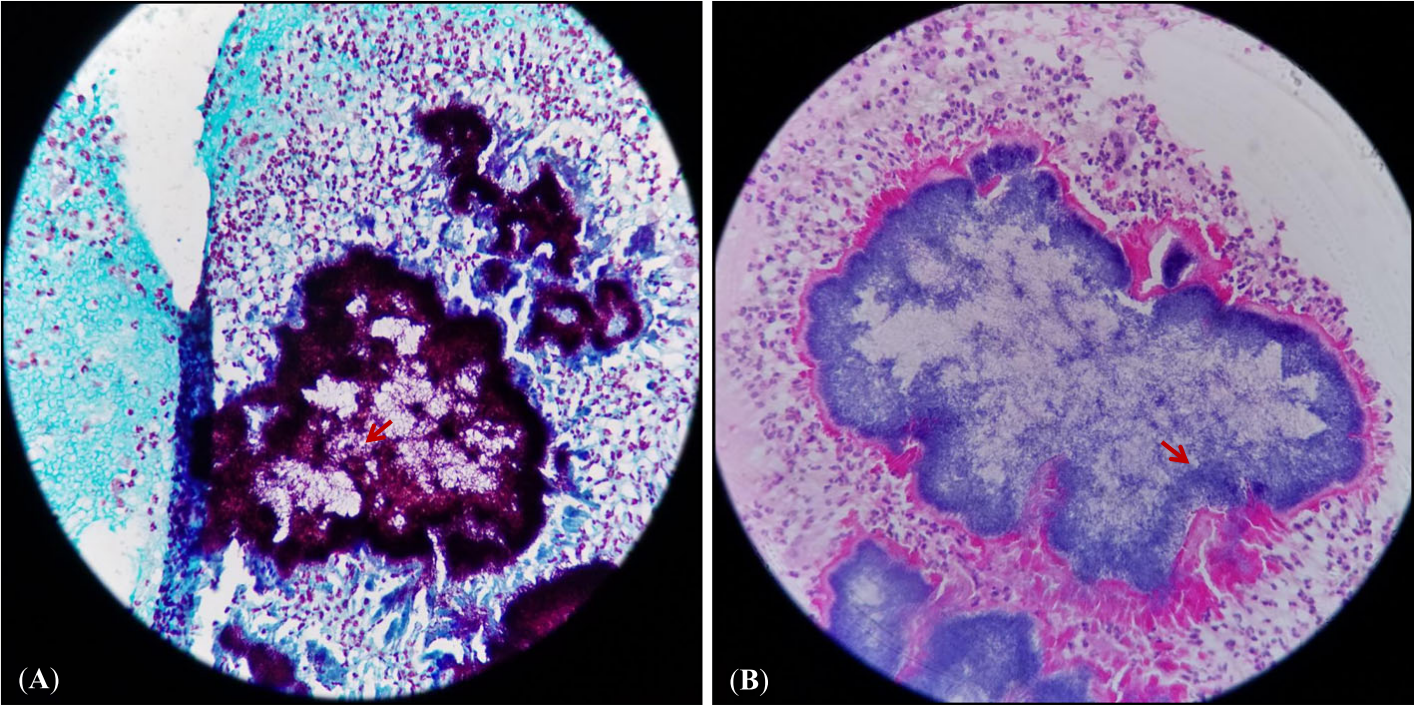
**a** Cutaneous biopsy of the left foot, presenting pseudofilamentous bacteria in Gram stain at objective × 40; **b** Skin biopsy of the left foot, demonstrating mycetoma grain stained by HES at objective × 40

**Fig. 3:**
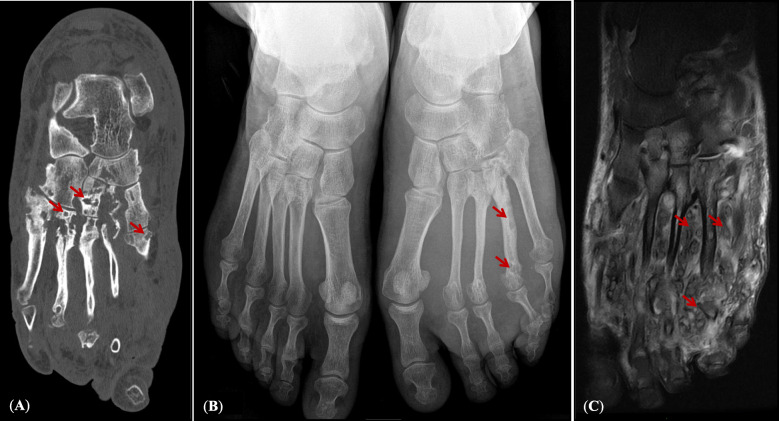
**a** Left foot tomodensitometry demonstrating metatarsals osteolysis and thickened soft parts; **b** Foot X-rays with thickened soft parts of the left foot and the 4th metatarsal osteolysis; **c** Soft tissue infiltration by bacteria and bone involvement of the left foot demonstrated using T2 sequence fat-sat pulses in MRI

Eighteen months later, the patient was referred with the same problem to Avicenne Hospital (Bobigny, France), for accurate identification of causative agent(s) and re-initiating an adapted treatment. Clinical diagnosis revealed 3 cm enlargement in tumefaction circumference, presence of 11 sinuses in plantar, 10 sinuses on the topside of the foot, and emission of white grains (Fig. 1). Direct microscopic examination of crushed grains showed neither mycelial nor actinomycete filaments. A deep tissue biopsy was performed for histopathological examination and the sinuses secretions including the grains were used for microbiological cultures and molecular analysis. The histopathological assessment showed pseudofilamentous Gram-positive bacteria (Fig. 2a), necrotic debris, granulomatous and polymorphic inflammatory infiltrates, confirming an actinomycetoma (Fig. 2b). Cultures on Loewenstein-Jensen, Sabouraud-chloramphenicol-gentamycin, and blood agar were negative after 3 months of incubation at 30 °C. The imaging assessments were performed again, confirming the extensive lesions of the initial assessment (Fig. 3).

**Fig. 4:**
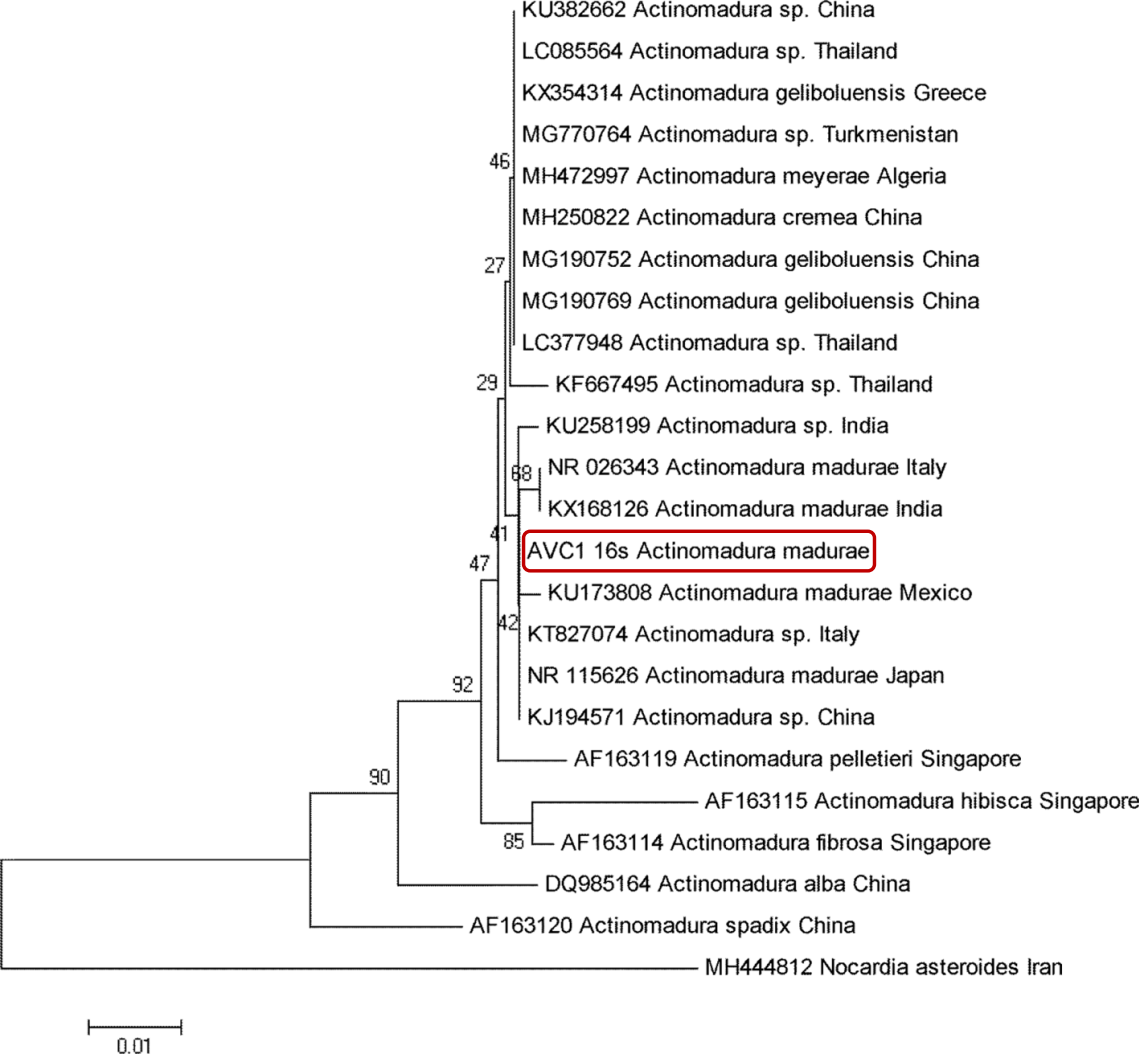
Neighbor-Joining (NJ) phylogenetic tree constructed based on 16S-rDNA gene sequence of *Actinomadura* species obtained in the present study (highlighted in red) together with those deposited in GenBank

Bacterial DNA in the grains isolated from the patient was extracted using bio-robot EZ1 and then subjected to a conventional PCR, targeting the 16S-rDNA gene with fD1 (fwd: AGAGTTTGATCCTGGCTCAG) and rp2 (rvs: ACGGCTACCTTGTTACGACTT) primers [9]. Obtained bilateral sequences were edited, aligned using BLAST, and identified as *Actinomadura madurae,* based on ≥99% identity with GenBank sequence (NR026343) and then deposited in GenBank with accession number NZ148818. In order to evaluate intraspecific variability, phylogenetic relationships, and polymorphisms within *Actinomadura* species, an inferred phylogenetic tree of *A. madurae* (identified in this study) together with GenBank sequences, was constructed based on the Neighbor-Joining method with bootstrap values, determined by 1000 replicates. This tree showed high congruence with the 16S-rDNA tree, since all taxa were concordantly clustered into the same species’ group (Fig. 4).

For treatment, trimethoprim/sulfamethoxazole (800/160 mg) was prescribed twice a day for 3 months with an expected duration of 1 year. Complete Blood Count (CBC) was routinely verified to ensure no overdose toxicity. Three months later, 2 cm reduction in left foot circumference was observed. Consequently, the treatment was continued until complete healing.

## Discussion and conclusions

Little is known about the mycetoma in Algeria. *Actinomadura madurae* was first described in 1894 by Vincent, as *Streptothrix maduruae*, based on several strains isolated from an Algerian case of Madura foot [10]. Afterward, other primitive cases of mycetoma caused by *A. madurae* were documented over 80 years ago [11, 12]. A case of mycetoma caused by *Streptomyces somaliensis* was later reported from the southern region of Atlas Mountain [13]. According to a retrospective investigation carried out from 1995 to 2005, 13 mycetoma cases were documented, from which five cases were actinomycetomas (caused by *A. madurae*, *S. somaliensis,* and *Nocardia asteroïdes*), three eumycetomas (caused by *Madurella mycetomatis*) and three without etiologic agent identification [14]. Diversity in causative agents of mycetoma in Algeria is geographic-dependent [14]. In the north, the humidity seems to be suitable for species such as *N. asteroïdes* and *M. mycetomatis,* while the southern arid/desert regions are favorable for the development of species such as *S. somaliensis* [14]. Although *Actinomadura* species is known as an agent of desert areas [15], we isolated it from a patient, originally from a humid region (880 mm rainfall), which supports the findings reported by Zait et al. [14]. Contrary to the little information found about the human actinomycetoma, the search for actinomycetes in the environment (water and soils) was performed in several parts of Algeria, in particular in the south and north-eastern regions, allowing to isolate several genera such as *Actinomadura, Saccharothrix,* and *Streptomyces* [16–19]. Imported cases of actinomycetoma have been already reported in European countries such as Italy [20], France [21] and Switzerland [22]. Nevertheless, some reports of autochthonous cases from Italy [23] and France [24] have been also documented, suggesting the local occurrence of actinomycetoma in Europe.

Diagnosis of the mycetoma and identification of etiological agents is a challenging issue, especially in non-endemic areas. Microbiological culture and histopathological analysis are the diagnostic tools used for the identification of causal agent(s) [7] but they possess some drawbacks, including failure in culture, difficulties in the direct examination or invasive biopsy [25]. Molecular analysis of bacterial species isolated from our patient revealed *A. madurae* as the etiologic agent with ≥99% identity with GenBank sequences. Therefore, molecular analysis can be considered as a reliable tool, asserting accurate identification of causal agents [26]. Regarding the increasing number of refugees coming from endemic areas to Europe, the knowledge improvement of the clinicians on mycetoma is essential, particularly in those regions with increasing immigration issues. Moreover, there is a lack of knowledge concerning the impact of climate change on the worldwide prevalence of mycetoma.

This case highlights the importance of early diagnosis and accurate identification of the causative organisms of mycetoma, which allows effective antibiotic therapy. Due to no improvement of our patient with amoxicillin as a first-line treatment, trimethoprim-sulfamethoxazole was prescribed, which led to an improvement in 3 months [27]. The latter is considered as the gold standard for actinomyces treatment, particularly in cases of bone invasion.

## Data Availability

Data sharing is not applicable to this article as no datasets were generated or analyzed during the current study.

## Abbreviation

MRI: Magnetic resonance imaging
